# Recent Advances in Olefin Metathesis

**DOI:** 10.3390/molecules21121751

**Published:** 2016-12-21

**Authors:** Georgios C. Vougioukalakis

**Affiliations:** Laboratory of Organic Chemistry, Department of Chemistry, National and Kapodistrian University of Athens, Panepistimiopolis, 15771 Athens, Greece; vougiouk@chem.uoa.gr; Tel.: +30-210-7274230

Olefin metathesis is one of the most significant developments of the last 20 years in the fields of organic chemistry, polymers synthesis, and materials science [1,2,3,4,5,6,7]. It offers a new exciting toolbox of synthetic methods and, therefore, has conditioned the ways chemists design new molecules. Equally importantly, metathesis has stimulated the preparation of numerous fascinating novel compounds and materials in both industry and academia. The recognition of the great influence of olefin metathesis to the art of chemical synthesis led to the awarding of the 2005 Nobel Prize in Chemistry to Yves Chauvin, Robert H. Grubbs, and Richard R. Schrock [8].

The goal of the Special Issue of *Molecules* entitled “Olefin Metathesis” was to discuss recent developments in all fields of metathesis. This Special Issue consists of five research and two review articles. In the first research article, Pitsikalis and co-workers report the statistical ring-opening metathesis copolymerization of norbornene and cyclopentene by Grubbs’ first-generation catalyst [9]. Moreover, Choudhury and Welker discuss the preparation of 2-silicon-substituted 1,3-dienes, containing non transferrable groups known to promote transmetallation, via Grignard chemistry and enyne metathesis [10]. These dienes participate in one pot metathesis/Diels-Alder reactions in regio- and diastereo-selective fashions. In another research work, Schatz and co-workers report a study of olefin metathesis in water and in air, improved by supramolecular additives [11]. This is achieved by commercially available water-immiscible precatalysts and sulfocalixarenes, which are used to boost the reactivity of the metathesis reaction by catalyst activation, improved mass transfer, and solubility of reactants in the aqueous reaction media. Additionally, Paraskevopoulou, Mertis, and co-workers discuss a study on the reactivity of the bimetallic Na[W_2_(μ-Cl)_3_Cl_4_(THF)_2_]•(THF)_3_ towards the polymerization of selected cycloolefins [12]. In the last research article of this Special Issue, Poater envisages the exchange of ruthenium *N*-heterocyclic carbene complexes by neutral or charged rhodium-based metathesis catalysts [13]. This study is based on density functional theory calculations.

The next article of the issue reviews the catalytic properties of metathesis ruthenium complexes bearing *N*-heterocyclic carbene ligands with stereogenic centers on the backbone [14]. Amongst others, Grisi and co-workers discuss the differences in catalytic behavior depending on the backbone configuration of symmetrical and unsymmetrical *N*-heterocyclic carbenes. The second review article, by Dragutan, Simionescu, and co-workers, summarizes the work carried out in the field of controlled “living” ring-opening metathesis polymerization for the preparation of iron-containing organometallic complexes, as well as the advanced applications of these hybrid materials [15].
